# Some Notes on the Early Art of Extracting Teeth

**Published:** 1889-10

**Authors:** Frederick Sleep


ARTICLE III.

### SOME NOTES ON THE EARLY ART OF EXTRACTING TEETH.

BY FREDERICK SLEEP, L. D. S., GLAS. & DUB.

The awe which an imperfect knowledge of the human frame and its workings must have created in early times reduced the practice of surgery rather to the treatment of dislocations, the repair of the osseous system after fractures, to the healing of lacerations of the more vascular structures, and to the common practice of midwifery. Other less frequent, but more dangerous accidents, were no doubt treated on the axiom that "no surgery is better than meddlesome surgery," and the patient being abandoned to the dispenser of mystery—faith—hope—expectancy and the vis medicatrix, did not a little to raise medicine to a creditable position. One law, coming through the Mosaic law-giver 1,400 years before Christ, was that "If a man smite on his man servant's tooth or his maid servant's tooth, he shall let him go free for his tooth's sake." Possibly "The learning of the Egyptians," which formed the greater part of Moses' knowledge, contributed much to the value set upon the dental organs, for from what we afterwards find mentioned by Herodotus (although nearly 1,000 years after), there is a great possibility that the teeth received more than a passing notice from the physicians even of the time of the Jewish law giver. The Jewish law of "tooth for tooth" pre-supposes to some method of extraction, probably gathered from former masters. "Oh, virgin daughter of Egypt," says Jeremiah, "in vain shall you use many medicines," Moreover, from the fact that Cyrus and Darius sent to Egypt for physicians, we may naturally conclude that it was the eradle of the arts and sciences. Referring again to Herodotus, we find that medicine in Egypt was practiced on a plan of separation, each physician treating a single disorder and no more, consequently the country swarmed with practitioners, some undertaking diseases of the eye, some of the head, others of the intestines, others of the teeth, and others diseases which were not local. They had laws to protect the nation against quackery, and *post-mortem* examinations were allowed to discover the cause of disease.

It has been declared that the different castes divided the body into divisions according to their different dignities. There is no proof of this however, as all the doctors were of the class Pastiphori.

The division of the practice of medicine too cannot be considered proof that the science had developed to such a very exalted position as to necessitate specialism, for the human body was said to be in possession of thirty-six demons, consequently it was necessary to invoke particular spirits according to the part affected. Nevertheless, that the division was capable of causing more attention to parts is a natural consequence, and that as a consequence dentistry began to be recognized as a science, there is little doubt. An inspection of ancient skulls of the Egyptians in Professor Flowers' collection at the Hunterian Museum certifies that decay was well known, and the collection of Joseph Mayer, F. R. S., of Liverpool, is said to possess specimens of artificial teeth carved in wood and ivory. On the authority, too, of a writer in the *British Medical Journal*, the Etruscan Museum of Corneto and the Etruscan Museum of the Vatican possess specimens both of artificial teeth and gold stoppings. Dr. Lauderer, of Athens (*Chemist and Druggist*), affirms that he has analyzed stopping taken from ancient Hellenic skulls, which proved to be made of volcanic ash, santorian earth and lime, and that another skull had a tooth filled with gold thread or leaf, those same skulls being collected by a friend of the writer's. Ancient history incontestably proves that artificial teeth were known and used in that country. Dr. Purland, also, some years ago declared he possessed a pivoted tooth from a mummy. Whilst, what is more to the point, Mr. Mummery avers that examination of early Egyptian skulls sufficiently testify to the fact that teeth were extracted.

He bases his verdict on the fact that the alveolar borders are sometimes rounded and not sharp as they would be had the roots been absorbed or gradually exfoliated. We must of course presume the rounded alveoli to indicate extraction of no very distant date prior to decease, else as far as my observations go, the alveoli would not differ in appearance.

As to the mode of operating or the instruments used, we are quite ignorant, there being nothing bearing resemblance to a surgical instrument in any museum I am acquainted with. Probably, the library begun by Ptolemy and finished by Cleopatra, would have contained likenesses and descriptions among the 700,000 volumes, but we are compelled to look to other quarters and depend a great deal on inference and deduction. One art depends on so many collateral ones for its progress, that we can but make a rough guess of the unknown from what we see of the known—the tools used in other departments of industry. That they were bronze is certain. Iron was said to have been discovered about Moses' time by the burning of Mount Ida, but the conquerors of Alexandria, 1300 years after, used generally (ferrum) bronze weapons, and there is no evidence that I can find that it was in anything like ordinary use among the Egyptians. That their instruments were rude is probable, but not a certainty, for many of the articles from Thebes demonstrate considerable artistic merit and mechanical skill.

The rise and progress of surgery among the Greeks was considerably hampered by the Athenian law that all who died orphans were commanded to be burned on the same day under a fine of 1000 drachma ; and moreover, by a superstitious reverence attached to the Greeks and Jews as nations, that it was profanation to touch the dead. Anatomy was therefore dependent on lower animals for exemplification, and consequently doubt and uncertainty was the result. Homer, however, mentions incisions and lenitives as the manner physicians cured wounds, and considering that he probably lived 900 years before Christ, we may consider medicine and surgery had taken root among the Greeks. The second son of Esculapius is credited as being the first Phlebotomist, having practiced on his future wife, who fell from a housetop, and thereby became both son-in-law to the King of Caria and possessor of a large dowry. Surely surgery was never better rewarded. The third Esculapius is, however, more entitled to the attention of the dentist, for the Greeks attributed to him the art of extracting teeth, and honored him accordingly. The history of every science and art is a history of progress; not alone was necessity the only stimulus, for honor of the most exalted character was showered on those who were the authors of such inventions as contributed to the welfare of humanity.

"Divine honors," remarks Bacon, "were rightly by antiquity attributed to *inventors*, but upon such as deserved well of their country only heroical honors were conferred." That medicine fell into worthy hands may be gathered from the following—"Artaxerxes (B. C. 425) sendeth to Histanes, the governor of Hellespont, this message: 'The fame and renown of the noble Hippocrates, of the race and lineage of Esculapius—bestow thou on him as much gold as he shall demand of thee, and whatsoever he shall have need of bestow it upon him in the most plentiful manner, and have a care to send him to me, for he shall be equal in honor and dignity to the greatest prince of Persia." History, however, records that the noble Father of Physic and Homer of his profession, returned answer even when threatened removal by force, that he served his country in preference to the foreigner. His words bear testimony of the greatest candor and honesty. He remarks that out of forty-two entrusted to his care only seventeen recovered. He expressed it as his opionion that the departments of surgery requiring special expertness should be entrusted to those who gave special attention to the particular branch. Physic is said to have gained by him more than it did by all who preceded and any one who came after him. His dental advice was that the teeth which are decayed and loosened be extracted, but that when neither decayed nor loosened, they be left behind and be cauterized, even although they cause violent pain. Aristotle, however, who lived quite one hundred years after, is the first who mentions the instrument for extraction, which he designates a forcep. His pupil, however, Heraclides (333 B.C.), as well as Herophilus, the first physician who dissected dead bodies (570 B.C.,) consequently practiced before Hippocrates, both relate cases of people dying from extraction of teeth; and Eristratus (257 B.C.), the grandson of Aristotle and a violent opposer to bleeding, cautiously in company with a great many down to modern times, speaks of the leaden tooth extractor in the temple of Delphos.

Celsus the Roman, who practiced during the Augustian era, and consequently whilst Jesus was on earth, was destined to form an epoch, and leave an impression on future surgery that should never be eradicated.

Some of the operations described by him are practical and nearly identical with thole of the present day. Yet great as was his ingenuity, discrimination and judgment as a surgeon, he directs us not to be in a hurry to extract teeth, but advises that stimulants be withdrawn and only soft food be taken; and if the toothache be violent, hot poultices to the belly and hot liquids to the mouth. He does, however, advise extraction when absolutely necessary, such as for ulcers of the gums. The operation is described thus: the gum must be detached from the tooth and the tooth repeatedly shaken till loosened, then taken out with the fingers or forceps, if not otherwise reached, taking care to fill with lint or lead to bear pressure of the blades of the instrument, which must not be pulled direct lest the thin bone (the alveoli) be broken, for the extraction of a firm tooth is, he avers, attended with the greatest danger; the jaw being sometimes dislocated, or the operation, if performed on the superior maxillary bone, may communicate a shock to the eyes or temples. Hæmorrhage he attributes to fractures of the jaw and advises resection of the fragments lest the jaw bone tumefy. Stumps he advises to be extracted with an instrument known as rizagra (root puller).

Arætœus of Cappadoria, but who practised at Rome about 100 A D., whose line of thought and style of writing has been so frequently recommended to the notice of the medical student, was a man bold, decisive and zealous, evidently described what he actually saw. He is credited with the remark that "The cause of the toothache is known only unto God." That he said so may be recorded in the writings of his contemporaries, but it certainly is not contained in his works. Being a pungent sentence it has been made use of by everybody who has written on ancient dentistry. Credulous he may have been from the mental atmosphere he lived in, but I think he was too wise a physician to lazily cast into the lumber of the unknown a disease because the trouble could not be immediately traced to its course. That superstition found in him an active opponent may be gathered from the following. He says, "I have seen people catch and drink the blood of the slain (criminal?) for certain diseases, but" remarks he, "I never yet heard of a cure, nor can we expect that the Creator would cure by such means."

Galen (131 A.D.) advised extraction, but preferred disintegrating the tooth with vinegar, &c. As he studied at Alexandria for some time we may assume he reflected the knowledge of the times. Even he, however, does not seem ever actually to have performed dissection, except on the simia and animals nearest to man; and as a proof as to how human anatomy was shut out from the world he revels in the opportunity he enjoyed of seeing and examining two skeletons at Alexandria, and advises students to go there to study the bones. He may be considered the last great Greek physician.

The splendor of Cæsar's conquests, together with the subjugation of Alexandria, transplanted medicine to Roman soil. Here the Egyptian and subtle Greek practised side by side on the foreigner—not that this was caused by a natural incapacity of the Roman, but by the national pride that caused the Patrician to shrink from practising themselves or educating their sons to a profession that was paid from the pockets of private individuals, and not from the public as a body. By-and-bye, however, the demand exceeded the supply, and slave and freedman contributed to swell the professional ranks. Among the foreigners was Aretœus, who practised there during the reign of Domitian, the last Cæsar So also did Galen practise there in his latter days. The first surgeon, however, who practised at Rome, one Archagatus (B.C.219) had a rough time of it; but, of course, things changed as civilization improved. He, however, assumed the title of Healer of Wounds, but found it better to decamp when the rabble changed it to *Executioner*. Things, however, changed for the better, for we find that tracheotomy and other operations were not uncommon during the reign of the Cæsars.

Aurelius, a physician of the Fourth Century, advised venesection and a cupping glass applied near an aching tooth, the bowels to be opened by a clyster, and the gums to be scarified. He extracted as a last resort, and did not consider that the operation removed the pain. Moreover, he says the eyes are almost always affected by extraction. Amid much that was trivial we find a great deal of sterling value that shows that experience and observation were not asleep. The Fifth Century, however, ushered in the destroyer in the persons of the Goths, Huns and Lombards, and medicine with all the other arts and sciences seemed almost destined to an eclipse, and with the exception of Ætius, who had studied at Alexandria, there was no one worthy of passing mention. Ætius is said to be the discoverer of the fact that the fifth pair of nerves supplied the teeth with nervous filaments.

The commencement of the Sixth Century saw things worse than before, and with the fall of Rome and the burning of the Alexandrian library, all that was noble seemed given over to Dioend and savagery, and if we except Paulus Ægineta, who probably practised in the Seventh Century, all is blank. This celebrated man had studied at Alexandria which even then lived on the fame of its former glory. He remarks that the teeth are pained without inflammation of the gums, sometimes from pain attacking the body of the teeth and sometimes from the nerve which enters them being affected, and he advises storax with opium put into the corroded part or to let the patient inhale henbane through a funnel. The antidote of Philo, also he esteemed a good remedy applied around the gum. This antidote, according to Hippocrates, was the liquid medicine of the skunk (animal?). Paul advised that loose teeth be fastened with aloes, galls, or alum boiled in wine. He with Ætius recommended spurge applied to the teeth for their removal without pain and operation, but Ætius speaks of red arsenic, which agent was doubtlessly able to effect the removal of the teeth and sometimes more too. (Dr. White, in an early volume of the *Cosmos*, declared he removed some teeth from a girl who would not submit to the operation of extraction, by the careful application of arsenic to the edge of the periosteum, and he states it was effected without pain or injury. The Chinese, too, have the credit of practising the same method from remote times.) Fortunately, however, for medicine and all that appertains to it, the trade between Mecca and Medina and Alexandria had introduced many ideas derived from a failing science, and luckily the works of the leading Greeks fell into the hands of the Arabians, and medicine, I feel no doubt, gained eventually by the transplantation. The first great light was destined to emanate from Rhazes, who lived about the middle of the Ninth Century. He has been called the Galen of Arabia, and he seems worthy of the apellation. He traveled far and wide in quest of knowledge, and history records the fact that he was the first who used chemicals in medicine. We are indebted to him for corrosive sublimate, mercurial ointment, various preparations of arsenic, the sulphates of copper, with saltpetre and borax. His system of dental surgery can scarcely be considered progressive, for he directs arsenic to be used as a remedy for habitual bleeding of the gums. Aching molar teeth he says should be cured by pouring boiling oil on them, and, like Hippocrates, he extols the cautery. "When teeth are broken," says he, "cauterise them; if loose, bind them to the stronger with a golden chain, and when extraction is made necessary, they are loosened if the carious cavity be filled with resin."

The Tenth Century produced another remarkable man in Avicenna, one whose works were destined to be text books and almost oracular for nearly 600 years. Avicenna seems to have extracted the same as the great one before him. He, too, declares that teeth may be taken out by the application of arsenic, worked up with the grease of the green frog. Moreover, he advised opening the radial veins, leeches to the gums, and cupping glasses below the chin for various diseases incidental to the oral cavity.

Avenzoar comes next; he lived in the Twelfth Century, and was known as the Experimenter, because of his careful studies of the powers of medicine. It is said that he lived 135 years, and obtained the highest honors in his country. He recommended arsenic for odontalgia, and advises the local application of not more than two grains. He says the patient must allow the saliva to dribble from the mouth during the application, and not run backwards, lest it be swallowed and do injury. Contemporary with Avenzoar was Albucas, known as Albucasis, the Arabian, a Spanish Moor, born near Cordova. He is remarkable as being the first who left drawings describing surgical instruments. He performed bronchotomy among other operations, and insists on the surgeon possessing a knowledge of anatomy. The actual cautery found a champion in Albucas, for he fills several chapters of his work with all that concerns the mystical of that potent agent. He might almost be considered a fire worshipper so rapturous does he become over its marvellous efficacy. Memory must ever record the fact that he was the first who ever used styptics for hæmorrhage, which has been more than once presented by the detractors of the memory of the illustrious Pare. It is, however, more than likely that Pare discovered the action of styptics spontaneously, for although he affected a knowledge of the ancients, it was only with difficulty and through the aid of friendly translators that he gained his knowledge of the ways and means of the ancients. The lover of the curious in the rise and progress of dental surgery and extraction, will be interested in the plates, descriptions, &c., appearing in his works. It must, however, be noticed in looking through the body of the work, I discover that the extracting instruments were not all made specially for the teeth, No. 2 on plate III. being used alike for extracting arrows and teeth; No. 3, on the same plate, being used in operations for closing fistula, &c. There is no translation of this eminent man's work that I am acquainted with, I therefore give as good a translation as time and practical life will allow me.

I shall take the liberty (over and above that of extraction) to insert the principles and practice advocated by this eminent pioneer of dental surgery, as I think he introduced an important practical era. Nor is dental surgery proper alone indebted to him, for he extended his practical views so far as to advocate a lost art—that of the manufacture of false teeth. I feel glad, therefore, of brushing away the cobwebs from this man's portrait, and holding him forth to the light of day as a remarkable man and worthy of our high estimation.

The great waves of thought which make an epoch arise but seldom, but when they do their influence is felt "to the last syllable of recorded time." Such men breathe over the dry bones and they live again—head and shoulders over their fellows rises their individualty. Their vision seems prophetic, and their actions savour of the miraculous to those living at the same time. Such were Hippocrates, Celsus, Avicenna, Albucas, and Hunter, whose words almost seem oracular to those who followed behind.

### CONCERNING THE EXTRACTION OF TEETH (*Albucas*).

It often happens that the pain of the teeth becomes insufferable because of the nerves and membranes which preside over them. You may at times make a cure with the exercise of considerable perseverance, or it may occur slowly by the teeth being exfoliated, not necessarily through age, when the teeth grow up and become loose.

Now, although cure may be rare, yet it is bad practice to extract where there is hope of salvation. When, however, the severity of the pain is caused by corruption in a large cavity, thorough extirpation of the tooth is indicated. Be careful, however, that you make a correct diagnosis of the offending member, for frequently the pain of the inferior tooth passes over to the healthy one, and that one is extracted, with no cessation of the misery till the real one is removed. Take heed, therefore that this happen not with you, lest the tooth be removed by the hand of a blood letter.

When, however, you are certain which is the painful tooth, it is needful that you scarify around the neck of the tooth with your lancet, but be careful in your cutting that you wound the gums no more than is requisite. Then move the tooth with your fingers, delicately at first for a time, until you have shaken it; then, after holding the head of the patient between your knees lest he shall move, then shalt seize hold of the tooth with the large forceps at a sound place, and then draw the tooth straight so you may not break it. If, however, it cannot be moved out, then take another instrument and insert it under the tooth carefully at different parts, and endeavor to move it up in the same careful manner as you have done before. If, however, the tooth is hollowed or rotten, then it behoves you that you pack the cavity with lint (panno), and strain it carefully with the point of the elevator that it may not be broken, whilst thou movest and strainest it with great endeavor with the forceps.

As before said, the scarificator should have been used well around the tooth so that it may be separated from the surrounding parts, and you should ever endeavor to pull straight and with a firm hand lest you break it, and the piece remaining, greater pain and trouble ensue than before. And beware you act not in the same manner as the ignorant bloodletters, who pull away swiftly at the teeth with the greatest impudence. Conceited men are anxious to be thought off-hand and decisive, although they have neither aptitude, scientific knowledge, nor prudence, to help them.

Too often, such men overawe weak minded men, whose miseries alas! are increased when their teeth are smashed and left in their jaws. At times, however, such fellows vary the operation by removing a piece of the jaw, and thus we see honest folk suffer at the hands of the rash and unskilful. After the extraction, the alveolus is to be washed out with wine and vinegar and salt, because a rush of blood may occur from the place, as often happens; now rub the place with zegum. If, however, that avail not, cauterise the place with the same instrument. The form of the light forceps with which the tooth is moved at first, should have long bills with short stout handles, so that you may not require a stronger pair when you operate on the upper teeth. And in like manner lest the points be bent, they may be shortened, and between the jaws you may grasp the tooth securely and with power, and sometimes too, the points are made like the bill of the bird known as the stork, and they are of a strong make.

### CONCERNING THE EXTRACTION OF FRACTURED TEETH AND BROKEN PIECES OF THE JAW.

Whenever in extraction, some part of the tooth remains which is broken, then it behoves you that you apply *cotum* with butter, one or two days, until the part is (mollified?) made soft, then insinuate the gestum or forceps, whose extremities partake of the appearance of the beak of a stork. The extremities of them may be made so as to curve inwards like an *alieralfigum*. If, therefore, the removal of the tooth follows not, then take heed you cut away the whole of the gum overlying and surrounding the root, and that you introduce an instrument which is called the little alalum, the form of which is appended.

The short extremity may be of middling thickness, and you must take care it is not broken. If, therefore, it passes through, it is well; but if it does not, help the operation with the other instruments of which these are forms. The form of the first has a triangular extremity, whilst the heavier form of the first triangular instrument is such as you have, figured first.

Moreover, the operation (before spoken of) may be even helped by an instrument having two branches, which is figured below. Also others of the instruments and steel tools for scaling, which are aforementioned, may be used. Again, you may operate with this instrument, which is fashioned like a large hook.

The triangular point is twisted, and stout, so that it may not be broken. It should be held properly and cir cumspectly in the hand; but understand that instruments for the teeth are of many shapes, and it is impossible to enumerate and describe them like other instruments which the operation demands of him, or the accident requires or calls for. Because, with such ailments and operations, there are so many peculiarities regarding which the greater number are not touched on, nor can the memory describe all the useful instruments, because of the diverse circumstances at times presented. If, however, a piece of the jaw or any other of the bones in the mouth is fractured or ulcerates, then operate above the offence with such instruments or forceps as may be most meet, as I have directed before concerning root extraction, and you may find the *gestum* handy, the form of which I now give, and it should be of sufficient strength to grasp the piece of bone, and not slip nor shift whilst the bone is being forced out. After which, treat the place with medicines most suited to the case.—*London Dental Record.*

